# Microgravity-Related Changes in Bone Density and Treatment Options: A Systematic Review

**DOI:** 10.3390/ijms23158650

**Published:** 2022-08-03

**Authors:** Ronni Baran, Markus Wehland, Herbert Schulz, Martina Heer, Manfred Infanger, Daniela Grimm

**Affiliations:** 1 Department of Biomedicine, Aarhus University, Ole Worms Allé 4, 8000 Aarhus, Denmark; 201709730@post.au.dk; 2 Department of Microgravity and Translational Regenerative Medicine, Otto von Guericke University, Universitätsplatz 2, 39106 Magdeburg, Germany; markus.wehland@med.ovgu.de (M.W.); herbert.schulz@med.ovgu.de (H.S.); manfred.infanger@med.ovgu.de (M.I.); 3 Research Group ‘Magdeburger Arbeitsgemeinschaft für Forschung unter Raumfahrt- und Schwerelosigkeitsbedingungen’ (MARS), Otto von Guericke University, Universitätsplatz 2, 39106 Magdeburg, Germany; 4 IU International University of Applied Sciences, 99084 Erfurt, Germany; martina.heer@iu.org; 5 Institute of Nutrition and Food Sciences, Nutritional Physiology, University of Bonn, 53115 Bonn, Germany

**Keywords:** spaceflight, microgravity, astronauts, cells, animals, bone loss, signaling pathways, countermeasures, pharmacology

## Abstract

Space travelers are exposed to microgravity (µ*g*), which induces enhanced bone loss compared to the age-related bone loss on Earth. Microgravity promotes an increased bone turnover, and this obstructs space exploration. This bone loss can be slowed down by exercise on treadmills or resistive apparatus. The objective of this systematic review is to provide a current overview of the state of the art of the field of bone loss in space and possible treatment options thereof. A total of 482 unique studies were searched through PubMed and Scopus, and 37 studies met the eligibility criteria. The studies showed that, despite increased bone formation during µ*g*, the increase in bone resorption was greater. Different types of exercise and pharmacological treatments with bisphosphonates, RANKL antibody (receptor activator of nuclear factor κβ ligand antibody), proteasome inhibitor, pan-caspase inhibitor, and interleukin-6 monoclonal antibody decrease bone resorption and promote bone formation. Additionally, recombinant irisin, cell-free fat extract, cyclic mechanical stretch-treated bone mesenchymal stem cell-derived exosomes, and strontium-containing hydroxyapatite nanoparticles also show some positive effects on bone loss.

## 1. Introduction

The length of a space mission and, thus, the amount of time space pilots are spending in orbit have been increased since humans have started conquering space. Space travelers are affected by numerous stressors in space, which include psychological factors such as isolation, psychosocial factors such as family disruption, human factors such as food restrictions, habitability factors such as lack of privacy, and physical factors such as radiation and microgravity (µ*g*) [1,2].

Microgravity is known to induce changes and health problems in the human body, such as cardiovascular, immune, or musculoskeletal health concerns [3,4,5]. One of the reasons for the problems in the musculoskeletal system is mechanical unloading caused by µ*g*. In addition, µ*g* leads to skeletal muscle atrophy and bone loss (BL), among other effects. BL can be seen as a reduction in bone mineral density (BMD). The µ*g*-induced reduction in BMD occurs at a fast rate of 0.5–1.5% per month in space [5,6,7].

Therefore, a systematic review was conducted to investigate to what extent this phenomenon resembles postmenopausal (PMP) osteoporosis (OP) on Earth, keeping in mind that the main cause/mechanism of postmenopausal osteoporosis is different from the reduction in BMD in µ*g*. For this purpose, recent findings on the mechanisms of µ*g*-related changes in bone density and possible treatment options are discussed. Furthermore, clinical trials are presented to complement in vitro with in vivo data. Overall, this review provides a current overview of the state of the art of the field of BL in space and also discusses possible implications for OP patients on Earth.

### 1.1. Postmenopausal Osteoporosis

The OP diagnostic criterion is a T-score ≤ −2.5 standard deviation below the mean for young healthy adults at the femoral neck, lumbar spine, or total hip by BMD testing [8,9,10]. PMP OP is known as primary OP type I and is caused most likely by reduced estrogen levels, which eventually lead to increased bone resorption (BRS) [8]. The bone mass reaches its maximum, named the peak bone mass, at the end of the bone maturation process around the age of 28–30 years. It is particularly dependent on a sufficient calcium intake, adequate vitamin D intake, genetics, and physical activity [11,12,13].

The increased BRS is closely related to hormonal changes, especially to the decline in the endogenous estrogen production at menopause. Estrogen has an inhibitory effect on osteoclasts (OCs); therefore, the deprivation of estrogen is associated with the loss of BMD. Deprivation of estrogen also contributes to increased urinary calcium loss and reduced intestinal calcium absorption, which is caused by calcium influx from the estrogen-deprived bones into the plasma and eventually results in reduced levels of parathyroid hormone (PTH) [14,15].

Following the European guidelines and the guidelines of the American Association of Clinical Endocrinologists (AACE), the treatment of OP can be divided into nonpharmacological and pharmacological strategies. The nonpharmacological strategy consists of lowering the risk of falling and optimizing the dietary intake. The risk of falling is lowered by physical exercise, reduction in consumption of alertness- and balance-altering medication, and home environment improvement, among others, while optimizing the dietary intake is achieved by ensuring that the patient consumes enough protein and calories, as well as calcium and vitamin D, which can be achieved with supplements [10,16]. A diet with high potential renal acid load might also increase urinary calcium excretion, and a diet high in alkaline precursors might reduce urinary calcium excretion (uCaV) and urinary N-telopeptide (uNTx), while increasing femoral neck, lumbar spine, and total hip BMD. In spaceflight, the effect depends very much on the exercise level/mechanical loading [17,18].

**Figure 1:**
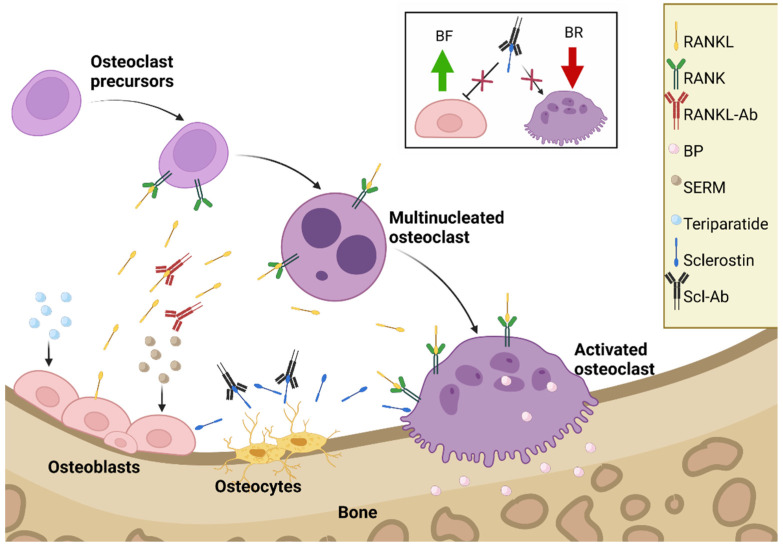
The treatment of menopausal osteoporosis. Abbreviations: RANKL, receptor activator of nuclear factor κβ ligand; SERMS, selective estrogen receptor modulator; Scl-Ab, antisclerostin antibody [10,16,19]. The insert shows the Scl-Ab’s dual mode of action in more detail. Created with BioRender.com (accessed on 27 July 2022).

Pharmacological treatment can be divided into antiresorptive (bisphosphonate (BP)), receptor activator of nuclear factor κβ ligand (RANKL) antibody, selective estrogen receptor modulator (SERM), antisclerostin antibody, and anabolic medication (see Figure 1) [10,16,19].

SERMs, such as raloxifene, reduce BL by binding to the estrogen receptor, where they act as an estrogen agonist or antagonist, depending on the tissue. BPs, such as alendronate, reduce BL by reducing OC recruitment and activity and increasing the apoptosis of OCs. RANKL antibodies, such as denosumab, reduce BL by binding to RANKL, which prevents OC activation. Teriparatide is an anabolic medication. It is the 1–34 N-terminal fragment of PTH; it induces bone formation (BF) and improves the skeletal architecture by increasing the activity and number of osteoblasts (OBs) [10,16].

Sclerostin, synthesized in osteocytes, inhibits bone formation. It is responsible to keep the balance between new bone tissue formation and resorption of old bone tissue. It exerts antianabolic effects on bone. The protein sclerostin binds to LRP5/6 receptors and inhibits wingless-related integration site (Wnt) signaling, which results in a reduction in bone formation [20]. Targeting sclerostin is important because of the protein’s special antianabolic action on bone formation. The humanized monoclonal antibody romosozumab (brand name Evenity), indicated for osteoporosis to reduce the risk of fractures, was approved by the Food and Drug Administration for use in the USA in April 2019 and in the European Union in December 2019. Recent rodent data showed that romosozumab and abaloparatide have additive effects when used as a countermeasure against disuse osteopenia in female rats [19]. 

Romosozumab exhibits a dual mode of action. Firstly, it enhances bone formation; secondly, it inhibits bone resorption. This mechanism is shown in Figure 1. The development of romosozumab offers a safe and efficacious new drug to treat osteoporosis in postmenopausal women and men. In addition, the drug may be interesting to be tested in space travelers [21].

### 1.2. Microgravity-Induced Bone Loss

**Figure 2:**
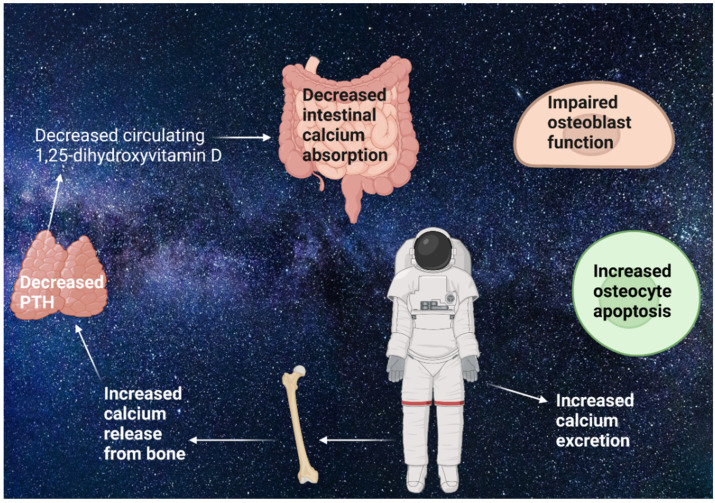
Mechanisms of microgravity (µ*g*)-induced bone loss: µ*g* releases calcium from bone, which suppresses the parathyroid hormone (PTH). Afterward, the suppressed PTH then lowers the circulating 1,25-dihydroxyvitamin D. This leads to decreased calcium absorption [24,26,27,28]. Additionally, osteoblast function is impaired, and osteocyte apoptosis is increased [5,32]. This results in unchanged or decreased bone formation and increased bone resorption, which leads to bone loss [22,23,24]. Created with BioRender.com (accessed on 27 July 2022).

The decreased mechanical loading of weight-bearing bones caused by µ*g* in space leads to BL in humans after more than 4 months of space travel [6,7]. This is a result of increased BRS and unchanged or decreased BF, which is seen in different human studies both in space and during bed rest [22,23,24] (see Figure 2). During the first 2 weeks of spaceflight, the BRS is especially exacerbated in humans [25,26,27]. Microgravity releases calcium from bone in humans, which suppresses PTH and lowers circulating 1,25-dihydroxyvitamin D, although 25-dihydroxyvitamin D concentrations are still adequate. This leads to decreased calcium absorption in humans [24,26,27,28]. The decreased BF is found to be a consequence of impaired OB function and increased osteocyte apoptosis which was detected in both in vivo and vitro studies, as well as in a study focusing on rats [5,29,30,31]. The latter used a total of 50 4-week-old male Wistar rats, which were not suspended (*n* = 25) or suspended by the tail for 2, 4, and 7 days (*n* = 25 total). The right tibia metaphyses were used for histomorphometric analysis, the right femurs were used for TUNEL assays, and the metaphyseal area in left femurs was used for Western blot and immunoprecipitation analyses. The authors could show that, in the samples, components of the antiapoptotic pathway were downregulated during unloading. These findings contribute to µ*g*-induced BL of 0.5–1.5% every month in humans [6,7,22].

Because of the harmful effects induced on the human organ system by µ*g*, it is necessary for a successful space mission to develop countermeasures (CMs) against the µ*g*-induced changes [33]. The CMs can be divided into (1) preflight CMs, which include physical exercise and physiological adaptation training, (2) inflight CMs, which include physical exercise, sensory–motor training, pharmaceuticals, and nutritional health, and (3) postflight CMs, which include rehabilitation [34]. The Advanced resistive exercise device (ARED), a cycle ergometer, and treadmill exercise are current inflight CMs, routinely used directly against µ*g*-induced BL [35]. The crewmembers on the International Space Station (ISS) are prescribed to do 2.5 h of exercise of personal preference per day for 6 days a week [36].

The ARED on board the ISS uses vacuum cylinders, making it effective in the µ*g* environment. It can provide up to 272 kg of concentric resistance, with a constant load through the range of motion of the body; during the eccentric phase, the ARED provides ~90% of the concentric load. Additionally, the ARED replicates the inertial characteristics normally experienced during gravity on Earth [37].

Crewmembers of the ISS can use the treadmill by a subject-loading device to fix themselves to the device. The subject loading makes the crewmembers able to exercise partially loaded, where the typical load is ~70%. The cycle ergometers have clipless pedals to fix the feet to the device and can provide up to 350 W load [36].

Furthermore, different drugs promoting bone formation have been studied. These so-called exercise pills include urolithin A and kartogenin. Urolithin A increased the exercise capacity and counteracted the decline of muscle function caused by age in rodents. Kartogenin was effective in promoting the differentiation of chondrocytes and the repair of cartilage in an in vitro study [38,39].

## 2. Materials and Methods

This systematic review was conducted following the Preferred Reporting Items for Systematic Reviews and Meta-Analyses (PRISMA) guidelines [40].

### 2.1. Eligibility Criteria

The inclusion criteria were defined by the PICO parameters, which include population, intervention, comparison, and outcome [41].

The inclusion criteria were humans ≥18 years old, animal models, or cell cultures involved in BRS or BF. This population needed to be exposed to the intervention of either µ*g* or µ*g* analogs, or possible CMs against µ*g*-induced BL. The population exposed to the interventions needed to be compared to healthy individuals, controls, or baseline. The controls included human, animals, and cells involved in BRS or BF.

The outcome needed to be either mechanism of µ*g*-induced BL or the effect of possible CMs against µ*g*-induced BL. The studies were not eligible if written in languages other than English or Danish, published before 1 January 2016, involving human individuals < 18 years old, or a systematic review, meta-analysis, or case report.

### 2.2. Information Sources

The literature search for this review was performed on the online repositories of PubMed (pubmed.ncbi.nlm.nih.gov) and Scopus (scopus.com), and the latest search was conducted on 28 March 2022.

### 2.3. Search

**Figure 3:**
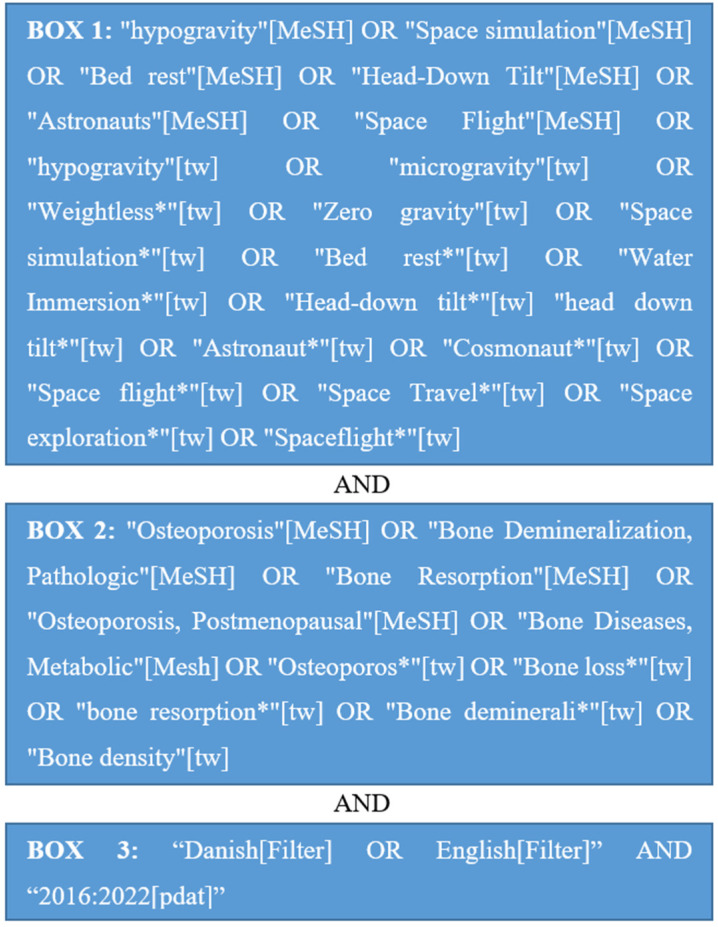
Search terms in PubMed.

Through a preliminary search in PubMed, relevant search terms were found as illustrated in Figure 3. Each search term in box 1 was combined with each search term in box 2 and all the filters in box 3 in several searches on 28 March 2022.

### 2.4. Study Selection

**Figure 4:**
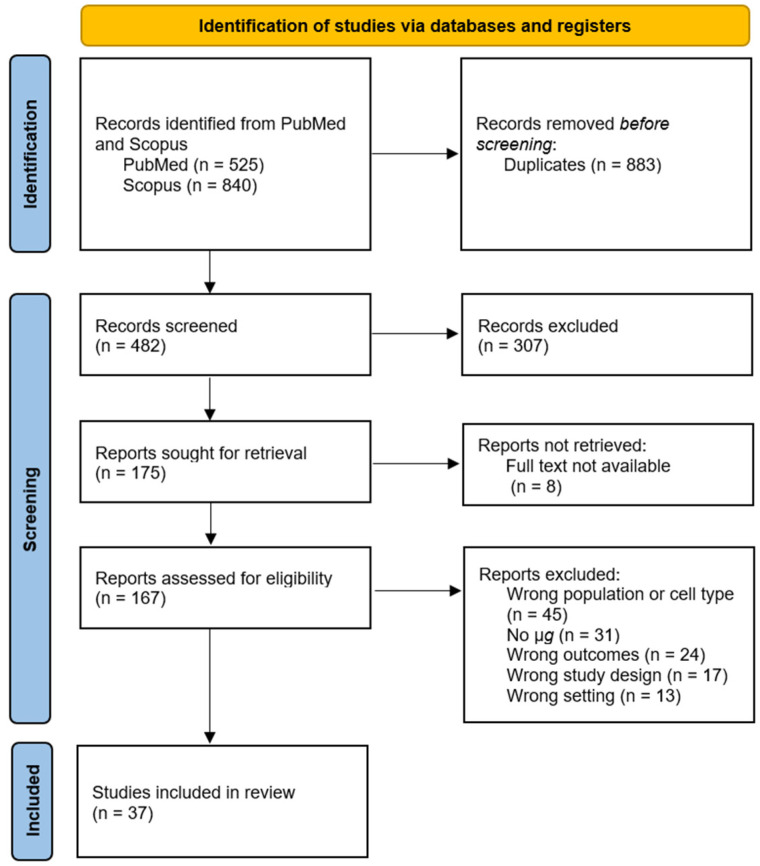
Flowchart of study selection.

The literature search resulted in a total of 1365 studies of which 883 were duplicates, leaving 482 studies. The reason for the duplicates is that each search term in box 1 of Figure 3 was combined with each search term in box 2 in several searches. After title and abstract screening for eligibility criteria, there were 307 studies excluded, leaving 175 studies. It was not possible to retrieve eight studies; thus, 167 studies remained. The full articles were screened for the eligibility criteria and their relevance to the objectives of this systematic review, which yielded 37 studies (see individual reasons for exclusion in the flowchart in Figure 4).

### 2.5. Data Collection Process

The author of this review developed a data extraction sheet, which was pilot-tested on three of the studies in case of any missing or redundant data. This was applied singlehandedly by the author of this review.

### 2.6. Data Items

The data obtained from the studies were as follows: first author, year of publication, population characteristics (number of participants, age, and gender), intervention characteristics (µ*g*/µ*g* analog and possible CMs), and outcome of either mechanism of µg-related changes in bone density or the effect of possible CMs for µ*g*-related changes in bone density.

### 2.7. Risk of Bias in Individual Studies

Studies in space are not only affected by µ*g*, but also affected by radiation, vibration during the launch from and return to Earth, and other environmental stressors in space, which also have the potential of inducing BL. This point cannot be excluded and may have impact on the findings. Low numbers of participants in space studies are also a problem since it makes it difficult to generalize the findings. Because of the risk of bias, these items were kept in mind when searching for studies.

### 2.8. Summary Measures

The outcomes measured were changes in BMD and other bone characteristics, as well as markers of bone cell activity.

### 2.9. Risk of Bias across Studies

Since the literature search only included published studies in English and Danish, there was a risk of both language bias and publication bias. These types of bias were kept in mind when searching for studies.

## 3. Results

### 3.1. Study Selection

The selection of the studies resulted in 37 studies as described in detail in Section 2.4. and in Figure 4.

### 3.2. Study Characteristics and Results of Individual Studies

An overview of the results of the mechanisms of µg-related changes in bone density are provided in Table 1, while the results of the possible CMs are given in Table 2.

BMD was decreased in humans by 0.9% after 6 months [42] and 1% per month in space [43]. The decrease in BMD in animals was 18–35.7% after 1 month [45,46].

Both markers of BRS [46,47,50,51,52] and BF [47,50] were elevated during µ*g* in humans and animals; meanwhile, several in vitro studies found inhibited OB differentiation markers (DMs) [54,56,57].

Additionally, sclerostin was increased in humans [51], while mRNA expression of *β-catenin* was decreased in OBs [54].

Resistive and locomotor exercise had an increasing effect on alkaline phosphatase (ALP) and attenuated the decrease in BMD during bed rest, respectively [60,61]. Resistive vibration exercise revealed an additional positive effect because it led to a greater proximal bone mineral content 6–24 months after bed rest compared to no exercise, which resistive exercise alone did not [61]. The positive effect of exercise on BL is supported by several animal studies [66,67].

BP, anti-RANKL, proteasome inhibitor (PI), pan-caspase inhibitor (PC-I), and interleukin-6 monoclonal antibody (IL-6 mAb) showed positive effects on BL [52,65,71,77], while SERMs and teriparatide did not reveal any effects on BL [65].

Additionally, recombinant irisin (R-irisin), cell-free fat extract, and injection of cyclic mechanical stretch-treated bone mesenchymal stem cell-derived exosomes (CMS) had protective effects on the bones [58,59,64,69,76]. 

Nanoparticles of hydroxyapatite (HA) loaded with risedronate were generated for bone-targeted drug delivery in ovariectomized rats (model for OP) [78]. This nanoparticle-based formulation was superior to risedronate sodium monotherapy in this model of postmenopausal osteoporosis. In addition, the zoledronic acid (ZOL)/HA nanoparticle-based drug formulation was tested in ovariectomized rats [79]. This formulation was highly effective in promoting BF. Moreover, the risedronate/zinc/HA-based nanomedicine revealed similar results. The findings showed that this nanomedicine had a therapeutic advantage over risedronate or risedronate/HA therapy for the treatment of osteoporosis in rats [80]. Furthermore, strontium hydroxyapatite (SrHA) and zoledronic acid (ZOL) nanoparticle-based drug formulation exerted therapeutic advantages over ZOL or SrHA monotherapy in experimental OP in rats [81].

Antioxidants (AO) only showed a positive effect on BL in animal and cell studies [72,75], but not in human studies [63].

### 3.3. Latest Clinical Trials

An overview of a selection of the latest clinical trials of CMs against and mechanisms of µ*g*-induced BL listed in clinicaltrials.gov as assessed on 19 April 2022 is given in Table 3. These studies primarily examined the effect of CMs on BMD and markers of bone cell activity in µ*g* analogs.

## 4. Discussion

### 4.1. Summary of Evidence

#### 4.1.1. Mechanisms of Microgravity-Related Changes in Bone Density

Earlier studies demonstrated a reduction in BMD of 0.5–1.5% per month in space [6,7]. This finding was supported by Burkhart et al., who reported a monthly reduction of 0.5–1% in BMD on the ISS in humans [43]. However, Bilancio et al. reported only a 0.9–1.4% decrease in total BMD after 6 months on the ISS in humans [42]. The rate of BMD reduction in mice seems to be higher because other studies found a reduction of 18–35.7% in BMD after 30 days in µ*g* [45,46]. This difference could be caused by biological differences, because the skeleton of mice keeps growing after puberty and lacks Haversian systems among other elements compared to humans [82]. The highest rate of age-related BL is found during the late perimenopause and beginning of the menopause, where the decrease of BL is up to 2.5% in the lumbar spine annually [83,84]. This indicates that the age-related rate of BL is substantially lower than the µ*g*-induced BMD reduction. However, the morphology of µ*g*-induced BL resembles the MP BL. BL caused by µ*g* reduced trabecular thickness and volume, and increased osteocyte apoptosis in the trabecular bone [45,46,52]. This resembles the MP BL, because the MP BL increases trabecular remodeling [14,85]. Sibonga et al. reported that, after a 6 month-stay in space, four of 10 astronauts had an incomplete recovery from µ*g*-induced loss of BMD 2 years after returning to Earth [44]. Interestingly, it was observed that only the trabecular BL, but not the cortical BL was recovered in mice 1 week after 4 weeks of spaceflight [48].

The µ*g*-induced decrease in BMD is caused by an elevated bone resorption. Several studies found elevated markers of bone matrix degradation and OC markers and activation in both humans and animals [47,50,51,52], which is supported by the 140% increase in BRS found by Gerbaix et al. [46]. However, markers of OBs were also elevated in several studies [47,50]. These findings suggest that both OC and OB activities are increased during µ*g*, but that the process of BRS must be greater than the BF due to the decreased BMD. In untreated PMP osteoporotic women, there was an increase in BRS markers reported, whereas the BF markers differed more between different studies [86,87,88,89].

The increased sclerostin in humans and the decreased mRNA expression of *β-catenin* in OBs during µ*g* suggest that the heightened bone turnover (BT) is caused by inhibition of the Wnt/β-catenin signaling pathway, leading to increased BRS and decreased BF [50,51,54,90]. However, numerous studies demonstrated that serum levels of sclerostin were significantly lower in PMP women with OP than in women without OP [91,92], which is different from the higher levels of sclerostin found during µ*g* [50,51]. The discrepancy in sclerostin levels could be a consequence of biological differences because the majority of the subjects in the µ*g* studies were male.

Several in vitro studies on cultured cells showed that the differentiation of OBs to osteocytes was inhibited, because OB DMs were decreased [54,56,57]. However, Cazzaniga et al. measured an increase in OB DMs after 4 days of µ*g* in human bone mesenchymal stem cells (bMSCs) [55]. This inconsistency could be attributed to differences in the length of µ*g* exposure and different cell types. To compare these data with findings in PMP women, we must focus on estrogen deficiency in cells, with studies showing that several OB DMs were decreased in estrogen-depleted bMSCs and pre-OBs [93,94]. These data suggest that the estrogen-depleted MP OP induces a decrease in OB differentiation, which is similar to several studies on µ*g*-induced BL.

#### 4.1.2. Possible Treatment Options for Microgravity-Induced Bone Loss

Both resistive exercise and locomotor exercise exhibited an increasing effect on the OB DM ALP and attenuated the decrease in BMD during bed rest, respectively [60,61]. Animal studies support the effectiveness of exercise on µ*g*-induced BL. Tibial compression during hindlimb unloading (HLU) had a protective effect on BL, while constrained dynamic loading increased bone volume fraction (BV/TV) [67], trabecular number and thickness, and OB DM [66].

Exercise is an important part of treatment of PMP OP [10,16]. Several studies on PMP women showed an increase in BMD after resistive and aerobic exercise [95,96,97,98].

The pharmacological treatment of MP OP is an important part of the therapy according to the European and AACE guidelines [10,16]. In addition, these treatments were tested in simulated µ*g* (s-µ*g*). BP and anti-RANKL inhibited the BRS and restored BMD in mice exposed to HLU close to mice in normal gravity in several studies [65,71]. Furthermore, 3-month-old male Wistar rats subjected to right hindlimb immobilization for 10 weeks to induce osteopenia were treated with zoledronic acid and alfacalcidol. This combination therapy was more effective than each drug administered as a monotherapy for the treatment of disuse osteoporosis [74]. However, SERM and teriparatide did not reveal any effect on BL [65], while BP, SERM, anti-RANKL, and teriparatide demonstrated a significant increase in BMD in MP women [99,100,101,102,103,104]. It should not be forgotten, however, that administration of BP and anti-RANKL can also cause some more or less severe side-effects. Overall, BP reduces fracture risk, but also induces changes in the bone material, which can reduce bone toughness [105]. More severely, BP and anti-RANKL are also linked to the development of osteonecrosis of the jaw, a condition which can lead to life-threatening complications [106,107,108]. The missing effect of raloxifene and teriparatide in µ*g* could be attributed to the biological difference between humans and mice. Additionally, the PI inhibited BRS, promoted BF, and induced an increase in BMD in mice exposed to HLU [65], while it decreased the area of resorbed bone in pre-OCs exposed to s-µ*g* [77]. Ovariectomy in animals is used as a model for PMP OP [109]. The PI increased the bone volume in ovariectomized animals, while it inhibited the OC formation and BRS in vitro, as well as stimulated OB differentiation [110,111]. PC-Is prevented the HLU-induced increase in osteocyte apoptosis, osteocyte RANKL expression, and endocortical resorption in mice [52]. PC-Is might have a positive effect on PMP OP. The OC differentiation was blocked by PC-Is in OCs [112], and the decreases in BV/TV, as well as trabecular number and thickness, caused by ovariectomy in rats were alleviated [113].

Furthermore, IL-6 mAb alleviates the µ*g*-induced BL by normalizing the BF and BRS, which leads to an increased BMD [73]. Meanwhile, another study only measured a decrease in the number of OCs [71]. This difference could be credited to different experimental protocols and strains of mice. Antibodies are already used in the treatment of MP OP, but not IL-6 mAb, which shows varying results. Some in vitro studies showed a suppressed development of OCs caused by IL-6 mAb [114,115]. Moreover, other studies, both in vitro and in vivo, showed no effect of IL-6 mAb on BRS, BL, or OC development [114,116,117]. The use of IL-6 mAb in the treatment of MP OP is still quite unknown, and further studies are necessary.

Pharmacological treatments are vital for future space exploration, especially because the pharmacodynamics and pharmacokinetics might be different in space [118,119]. Absorption of paracetamol showed both slower and faster peak concentration during µ*g* in different studies [118,119,120], while metabolism through P450 enzymes in rats was reduced by 0–50% after 7–14 days of spaceflight [118,121,122,123].

Furthermore, the effect of different supplements on µ*g*-induced BL was evaluated. R-irisin injection on mice during HLU prevented BL. There was no measurable loss of BMD, and R-irisin recovered the bone mass through attenuation of the OC inhibitor osteoprotegerin and increased activity and differentiation of OBs [58,64,76]. In ovariectomized mice, R-irisin prevented trabecular BL and induced greater BMD, while the number of OBs was increased, and the number of OCs was decreased in parallel [124]. Additionally, R-irisin returns the serum levels of osteocalcin, ALP, and tartrate-resistant acid phosphatase 5b in ovariectomized rats back to the same serum levels as non-ovariectomized rats [125]. Addition of SCHN to bMSCs had a protective effect on µ*g*-induced reduction of ALP activity and a promoting effect on the deposition of hydroxyapatite crystals [59]. Strontium ranelate is already used in OP treatment, where it showed a significant increase in BMD on PMP OP [126,127]. The injection of cell-free fat extract also has a positive effect on BL, because it alleviates the HLU-induced decrease in BV/TV, trabecular number, and cortical thickness [69]. Furthermore, the injection of CMS in mice exposed to HLU increased BMD, BV/TV, cortical thickness, and trabecular thickness and number, while reducing the number of OCs [70]. However, pulse-based diet and dietary protein and alkaline supplement did not show any effect on BL in humans [51,62].

Application of AO had an effect in studies on cells and animals. Both nutraceuticals and polyphenols have an elevating effect on ALP activity in OBs exposed to s-µ*g* [72,75]. The reduced BL in rats could be due to alleviation of the µ*g*-induced inhibition of Wnt/β-catenin pathway, which enhances BF, because ALP, N-terminal propeptide of type 1 procollagen, and β-catenin are increased [72]. However, no significant effect in humans on BL, who already consumed sufficient amounts of polyphenols, was found [63], which questions the use of AO against µ*g*-induced BL. AO for MP women also showed variable results. A study found no effect of vitamin C in PMP women [128]. However, other studies showed that the polyphenol resveratrol alone and curcumin combined with alendronate increased BMD in PMP women [129,130]. Here, the question remains whether the respective test subjects received adequate amounts of antioxidants before starting the study. These findings suggest that AO might have, under certain circumstances, an effect as a supplement to the treatment as advised in the European and AACE guidelines [10,16]. 

### 4.2. Strengths and Limitations

There were several strengths of this review. The data were individually searched and collected from PubMed and Scopus, and then systemically and manually examined on the basis of PICO parameters. Another strength is that the results were based on the latest data from 2016 onward, and we did not use systematic reviews or meta-analyses, where data are interpreted by additional authors, or case reports in the results.

There were also limitations to this work. This systematic review focused on the mechanisms behind µ*g*-induced BL and the possible treatment thereof on humans, but this review used studies examining animals and cell cultures, as well as studies examining s-µ*g*, which cannot replicate human studies in space. Nevertheless, the use of animals, cell cultures, and s-µ*g* have shown to be great alternatives to human studies in space [131]; therefore, this review argues that studies of animals and cell cultures and studies using s-µ*g* are justified in this case. Furthermore, the studies on CM in real µ*g* were performed on cells and not any on animals or humans. Since pharmacological treatments can be different in space [118,119,120,121,122,123], this may also be a limitation.

## 5. Conclusions

Space travelers experience µ*g*, which enhances BL by 0.5–1.5% and reduces BMD per month in space. Many, but not all studies indicated an elevated BF and a greater increase in BRS, which led to increased BT and enhanced BL. One reason for this finding is, among others, the inhibition of the Wnt/β-catenin signaling pathway. Space travels will be more common in the future. Therefore, the enhanced BL needs to be alleviated so that it does not limit the possibility of space travel. To counteract the µ*g*-induced BL, numerous CMs have been proposed. Firstly, adequate nutrient intake, particularly sufficient amounts of energy, protein, calcium, and vitamin D and a diet high in alkaline precursors, might reduce bone loss. Secondly, different types of exercise showed a positive effect on BF and BMD. Thirdly, pharmacological treatment with BP, anti-RANKL, PI, PC-I, and IL-6 mAb decreased BRS and promoted BF, which in turn induced an increase in BMD. However, SERM and teriparatide did not reveal a significant effect on BL. Additionally, injection of r-irisin, cell-free fat extract, and CMS, as well as the addition of SCHN, showed positive effects on BL. However, AO and other supplements delivered various results. Although, several CMs revealed promising results, the results are still too variable and the number of replications for any human study are still too small to propose definitive guidelines for space travels and long-term space exploration.

Taken together, more research is needed to expand the current knowledge of the mechanisms of µ*g*-induced BL. Studies on BL in space can be favorable and support the treatment of osteoporosis on Earth.

## Figures and Tables

**Table 1:** The mechanisms of µ*g*-related changes in bone density.

Author and Publication Year	Population	Intervention	Outcome
**Real microgravity in humans**			
Bilancio et al., 2019 [42]	1 male (52 years) and 1 female (37 years)	6 months on ISS	Compared to BSL: Decrease of 0.9 to 1.4% in total BMD; Increase of 27 to 116% in urine calcium/creatinine ratio.
Burkhart et al., 2020 [43]	1 female and 16 males (45 ± 4 years)	4–7 months on ISS	Between 4.6% and 6.1% decline in vertebral volumetric BMD (*p* < 0.05) compared to preflight. Vertebral volumetric BMD decreased 1% and lumbar spine BMD decreased 0.5% per month.
Sibonga et al., 2020 [44]	10 humans (gender N/A, 48 ± 5 years)	157 ± 21 days on ISS	5 of 10 astronauts had an incomplete recovery of BMD 1 year after return to Earth, which persisted in 4 of 5 astronauts after 2 years compared to BSL.
**Real microgravity in animals**			
Berg-Johansen et al., 2016 [45]	14 male C57BL/6 mice (19–20 weeks)	SG (*n* = 6): 30 days in low Earth orbit CG (*n* = 8): normal gravity	SG showed 20% reduced BV/TV (*p* < 0.05), 18% reduced BMD (*p* < 0.05), and 14% reduced trabecular thickness (*p* = 0.001) compared to CG. No significant difference in the trabecular number or trabecular spacing.
Gerbaix et al., 2017 [46]	22 C57/BL6 male mice (2 months)	SG (*n* = 10): 30 days in space CG (*n* = 12): normal gravity	SG decreased femur BV 64% and vertebrae BV 35.7%. SG increased bone resorption 140% and empty lacunae 344%. No bone recovery in SG 8 days after landing despite normalized OC activity.
Chatani et al., 2016 [47]	Medaka fish larvae in stage 39	SG (*n* = 3–9, depending on the anaylsis): 8 days on the ISS CG (*n* = 3–16, depending on the anaylsis): normal gravity	SG had significantly enhanced osterix, osteocalcin, TRAP5, and matrix metallopeptidase-9.
Von Kroge et al., 2021 [48]	C57BL/6N male mice (8–9 weeks)	SG (*n* = 5): 4 weeks in space CG (*n* = 5): normal gravity	After 4 weeks in space, BV/TV, cortical thickness, trabecular number, and thickness significantly decreased. This bone loss was only recovered in trabecular bone, and not in cortical thickness.
**Simulated microgravity in humans**			
Bonnefoy et al., 2022 [49]	20 male (34 ± 8 years)	SG: 60 days HDBR + antioxidant CG: 60 days HDBR	Compared to BSL: Decreased bone density of 1.4% (*p* < 0.0001) and BV/TV of 1% (*p* < 0.05).
Bemben et al., 2021 [50]	6 males and 5 females (25–50 years)	30 d HDBR	Compared to BSL: Increase in sclerostin, TRAP5, P1NP, and calcium. Decrease in total hip BMD and PTH. Women had a greater decrease in total hip BMD and increase in TRAP5 than men.
Buehlmeier et al., 2017 [51]	24 males (SG1 + SG2 ~60 years, SG3 ~23 years)	SG1 (*n* = 8): 14 days bed rest SG2 (*n* = 8): 14 days bed rest + CD SG3 (*n* = 7): 14 days bed rest	Urinary N-telopeptide of type I collagen (*p* < 0.05), urinary C-telopeptide of type I collagen (*p* = N/A), and sclerostin (*p* < 0.05) increased during bed rest in all groups compared to BSL.
**Simulated** **microgravity in animals**			
Cabahug-Zuckerman et al., 2016 [52]	24 C57BL/6J male mice (4 months)	SG1 (*n* = 6): 14 days HLU SG2 (*n* = 6): 14 days HLU + PC-I SG3 (*n* = 6): PC-I CG (*n* = 6): normal gravity	After 5 days: Increased RANKL-producing osteocytes and osteocyte apoptosis yjtrrfold in cortical bone and fourfold in trabecular SG1 compared to CG (*p* < 0.05). After 14 days: Only the increased osteocyte apoptosis in trabecular bone and RANKL-producing osteocytes remained significantly elevated threefold in SG1 compared to CG.
Chowdhury et al., 2016 [53]	61 male rats (3–18 rats per SG and CG, age N/A)	SG1: 2 weeks HLU SG2: 2 weeks X-ray IR SG3: 2 weeks HLU + X-ray IR CG: normal gravity	Significantly decreased BMD in distal femur and proximal tibia in SG1 and SG3 compared to CG (*p* < 0.05), but BMD was not significantly decreased in SG2.
Chen et al., 2021 [54]	12 C57/BL6 mice (2 months)	SG (*n* = 6): 4 weeks HLU CG (*n* = 6): normal gravity	MicroRNA-138-5p was upregulated during s-µ*g* in SG compared to CG (*p* < 0.01).
**Simulated** **microgravity in vitro**			
Chen et al., 2021 [54]	Murine pre-OB cells MC3T3-E1	SG: 12 h RPM CG: normal gravity	Compared to CG: MicroRNA-138-5p decreased *ALP* 93.1% and *collagen type 1 α-1* 64.9% in SG (*p* < 0.01). MicroRNA-138-5p decreased protein and mRNA expression of β-catenin in SG.
Cazzaniga et al., 2016 [55]	Human bMSCs	SG: 4 days RPM CG: normal gravity	SG show a significant upregulation of heat shock protein 60, heat-shock protein 70, superoxide dismutase 2 and cylclooxygenase 2, and a significant increase in RUNX2 and osterix compared to CG.
Chen et al., 2016 [56]	Rat bMSCs	SG: 2 days clinostat CG: normal gravity	Clinorotation significantly depolymerizes F-actin, and this hinders “transcriptional coactivator with PDZ-binding motif” nuclear. Furthermore, s-µ*g* inhibited ALP and RUNX2.
Zhang et al., 2020 [57]	Murine pre-OB MC3T3-E1	SG: 72 h clinorotation CG: normal gravity	Expression of all but *microRNA-30a* in the microRNA-30 family is upregulated in s-µ*g*. This is negatively correlated with expression of *RUNX2*, osteocalcin and *ALP*, which decreased during s-µ*g*.

Abbreviations: ISS: International Space Station, BSL: baseline, BMD; bone mineral density, N/A: not available, SG: study group, *n*: number of individuals, CG: control group, BV/TV: bone volume fraction, BV: bone volume OC: osteoclast, TRAP5: tartrate-resistant acid phosphatase 5b, HDBR: head-down tilt bed rest, P1NP: N-terminal propeptide of type 1 procollagen, PTH: parathyroid hormone, CD: cognitive training, and diet protein and alkaline, HLU: hindlimb-unloading, PC-I: pan-caspase inhibitor, RANKL: receptor activator of nuclear factor κβ ligand, IR: irradiation, s-µ*g*: simulated microgravity, OB: osteoblast, RPM: random positioning machine, ALP: alkaline phosphatase, bMSC: bone mesenchymal stem cell, RUNX2: RUNX family transcription factor 2.

**Table 2:** Possible CMs for µ*g*-induced bone loss and their effects.

Author and Publication Year	Population	Intervention	Outcome
**Real microgravity in vitro**			
Colucci et al., 2020 [58]	System 1: OC/OB System 2: OC/EC System 3: OC/OB/EC	SG1: 14 days ISS + R-irisin SG2: 14 days ISS CG1: normal gravity CG2: normal gravity + R-irisin	R-irisin in SG1 prevented the downregulation of RUNX2, osterix, activating transcription factor 4, osteoprotegerin, and *Collα1* caused by microgravity.
Cristofaro et al., 2019 [59]	Human bMSCs	SG: 88 h ISS + SCHN CG: normal gravity	SCHN exhibited a protective effect in SG on the reduced ALP activity caused by microgravity compared to CG (*p* < 0.05).
**Simulated** **microgravity in humans**			
Cavanagh et al., 2016 [60]	6 male and 6 female (30.2 ± 6.8 years)	SG (*n* = 6): 84 days HDBR + LE CG (*n* = 6): 84 days HDBR	BMD in the intertrochanteric and total hip regions was decreased in CG, but not in SG compared to baseline. The BMD loss was higher in CG than in SG.
Belavý et al., 2016 [61]	24 males (32 ± 10.6 years)	SG1 (*n* = 7): 60 days BR + vibration RE SG2 (*n* = 8): 60 days BR + RE CG (*n* = 9): 60 days BR	In SG1 and SG2 bone-specific ALP increased significantly more than in CG. SG1 also showed a greater proximal femur bone mineral content 6–24 months after BR compared to CG (*p* = 0.01). There was no significant difference on sclerostin and dickkopf-1 proteins.
Gao et al., 2019 [62]	2 males and 4 females (30 ± 12 years)	Cross-over study: 4 days of BR with either PBD or hospital diet separated by 30 days	The PBD attenuated the increase of urinary N-telopeptide of type I collagen to 33% ± 20% from 89% ± 75% during hospital diet. PBD had no effect on BMD compared to hospital diet.
Buehlmeier et al., 2017 [51]	24 males (SG1 + SG2 ~60 years, SG3 ~23 years)	SG1 (*n* = 8): 14 days BR SG2 (*n* = 8): 14 days BR + CD SG3 (*n* = 8): 14 days BR	No systematic difference between the SGs.
Austermann et al., 2021 [63]	20 males (3 ± 8 years)	SG (*n* = 10): 60 days HDBR + antioxidant CG (*n* = 10): 60 days HDBR	Antioxidant supplement had no effect on bone resorption or formation.
**Simulated** **microgravity in animals**			
Cabahug-Zuckerman et al., 2016 [52]	24 C57BL/6J male mice (4 months)	SG1 (*n* = 6): 14 days HLU SG2 (*n* = 6): 14 days HLU + PC-I SG3 (*n* = 6): PC-I CG (*n* = 6): no intervention	The PC-I used in SG2 prevented the HLU-induced increase in osteocyte apoptosis, osteocyte RANKL expression, and endocortical resorption in both cortical and trabecular bone compared to SG1.
Colaianni et al., 2017 [64]	32 C57BL6 male mice (2 months)	SG1 (*n* = 8): 4 weeks HLU + R-irisin SG2 (*n* = 8): 4 weeks HLU SG3 (*n* = 8): R-irisin CG (*n* = 8): vehicle injection	Compared to CG: R-irisin prevented loss of cortical or trabecular BMD in SG1. R-irisin induced the recovery of bone mass through attenuation of osteoprotegerin; thus, the RANKL/osteoprotegerin ratio was the same in SG1 as CG. R-irisin also inhibited the decrease in *ALP* and *Collα1* mRNA expression caused by simulated microgravity in SG1.
Ding et al., 2021 [65]	42 C57BL/6J male mice (12 weeks)	SG1 (*n* = 6): 28 days HLU + alendronate SG2 (*n* = 6): 28 days HLU + raloxifene SG3 (*n* = 6): 28 days HLU + teriparatide SG4 (*n* = 6): 28 days HLU + Anti-RANKL SG5 (*n* = 6): 28 days HLU + bortezomib CG1 (*n* = 6): normal gravity CG2 (*n* = 6): 28 days HLU	SG1 and SG4 reduced urinary C-telopeptide of type I collagen compared to CG2 (*p* < 0.05) and restored BMD close to CG1. SG5 reduced urinary C-telopeptide of type I collagen (*p* < 0.05) and enhanced P1NP compared to CG2 (*p* < 0.05), which increased BMD and strength compared to CG2 (*p* < 0.05). SG2 had no effect on bone loss. SG3 only stimulated cortical bone formation.
Han et al., 2018 [66]	48 WTC57BL/6J mice (2–3 months) 32 KOC mice (2–3 months)	SG1 (*n* = 16): 4 weeks HLU + CDL SG2 (*n* = 16): 4 weeks HLU + KOC SG3 (*n* = 16): 4 weeks HLU + CDL + KOC CG1 (*n* = 16): 4 weeks HLU CG2 (*n* = 16): normal gravity	Increased BV/TV, trabecular number and thickness, ALP activity, osteocalcin content, and mRNA level of *bone morphogenetic protein-2*, *Collα1*, *ALP*, and *osteocalcin* in SG1 compared to CG1, but this effect was even bigger in SG3 with combined CDL and KOC. Osteoprotegerin increased, while RANKL level decreased in SG3 compared to CG1; however, in SG1, only osteoprotegerin increased compared to CG1.
DeLong et al., 2020 [67]	35 C57BL/6J male mice (16 weeks)	SG (*n* = 15): 3 weeks HLU + TC CG1 (*n* = 10): 3 weeks HLU CG2 (*n* = 10): 3 weeks TC	In SG, a 2% loss in cortical thickness and 15% loss in trabecular BV/TV was observed compared to 6% and 50% corresponding losses in CG1. This protective effect did not influence the cortical bone at lower strained distal shaft.
Yang et al., 2021 [68]	36 C57BL/6J male mice (8 weeks)	SG (*n* = 6): 4 weeks HLU + SMF CG1 (*n* = 6): 4 weeks GMF CG2 (*n* = 6): 4 weeks SMF CG3 (*n* = 6): 4 weeks HLU CG4 (*n* = 6): 4 weeks HLU + GMF	SG had an increased BV/TV, trabecular number, connectivity density, cortical area, and femoral bone mineral content compared to CG4 (*p* < 0.05). Additionally, TRAP5 decreased during SMF compared to CG4 (*p* < 0.05).
Xu et al., 2022 [69]	18 C57BL/6 male mice (8 weeks)	SG (*n* = 6): 4 weeks HLU + CEFFE CG1 (*n* = 6): normal gravity CG2 (*n* = 6): 4 weeks HLU + PBS	Compared to CG2: CEFFE in SG increased BV/TV and trabecular number and cortical thickness. Additionally, the number of empty lacunae was reduced by CEFFE.
Xiao et al., 2021 [70]	40 C57BL/6J male mice (6 months)	SG1 (*n* = 10): 28 dats HLU + CMS SG2 (*n* = 10): 28 dats HLU + STE CG1 (*n* = 10): normal gravity + PBS CG2 (*n* = 10): 28 days HLU + PBS	Compared to CG2: CMS increased the BMD, BV/TV, cortical thickness, and trabecular thickness and number. CMS decreased trabecular spacing, number of OCs per field, and percentage of OC surface per bone surface. STE only increased BV/TV, as well as trabecular thickness and number, and decreased trabecular spacing.
Wakabayashi et al., 2020 [71]	32 ddY male mice (8 weeks)	SG1 (*n* = 8): 4 weeks HLU + IL-6 mAb SG2 (*n* = 8): 4 weeks HLU + alendronate CG1 (*n* = 8): normal gravity + vehicle CG2 (*n* = 8): 4 weeks HLU + vehicle	Compared to CG2: IL-6 mAb in SG1 reduced number of OCs per bone perimeter compared to CG2 (*p* < 0.05). Alendronate in SG2 increased BV/TV and trabecular number, while decreasing number of OCs per bone perimeter in both femur and tibia (*p* < 0.05).
Diao et al., 2018 [72]	50 SD male rats (6 weeks)	SG1 (*n* = 30): 30 days HLU + polyphenol SG2 (*n* = 10): 30 days HLU CG (*n* = 10): normal gravity	SG1 compared to SG2: SG1 alleviated the rise of bone surface/bone volume ratio and decreased ME, BMD, and BV/TV, but it differed from CG. SG1 increased ALP, P1NP, and expression of *RUNX2*, *Collα1*, *ALP*, *osteonectin*, *osterix*, *osteocalcin*, and *β-catenin*.
He et al., 2020 [73]	32 C57BL/6J male mice (10 weeks)	SG1 (*n* = 8): 4 weeks HLU SG2 (*n* = 8): 4 weeks HLU + IL-6 mAb CG1 (*n* = 8): normal gravity CG2 (*n* = 8): normal gravity + IL-6 mAb	Increased BMD, BV/TV, trabecular thickness, trabecular number, stiffness, and ultimate load in femur in SG2 compared to SG1 (*p* < 0.05). Serum osteocalcin and mRNA expression of *ALP*, *osteocalcin* and *TRAP5* increased, while RANKL/osteoprotegerin ratio decreased in SG2 compared to SG1 (*p* < 0.05). All these factors were normalized compared to CG1.
Khajuria et al., 2015 [74]	30 male Wistar rats (12 weeks)	SG1 (*n* = 6): 20 weeks RHLI SG2 (*n* = 6): 10 weeks RHLI+ 10 weeks RHLI/ZOL SG3 (*n* = 6): 10 weeks RHLI+ 10 weeks RHLI/ALF SG4 (*n* = 6): 10 weeks RHLI+ 10 weeks RHLI/ALF/ZOL CG (*n* = 6): nonimmobilized control	The combination of ZOL + ALF was more effective in decreasing bone porosity, in improving the mechanical strength of the femoral midshaft, and in improving dry bone and ash weights than the respective monotherapies.
**Simulated** **microgravity in vitro**			
Cristofaro et al., 2019 [59]	Human bMSCs	SG: 88 h RPM + SCHN CG: normal gravity	SCHN had a promoting effect in SG on the deposition of hydroxyapatite crystals compared to CG (*p* < 0.05).
Braveboy-Wagner et al., 2022 [75]	7F2 murine OBs	SG: 6 days RPM + nutraceuticals (curcumin, carnosic acid, and zinc) CG: 6 days RPM	Compared to CG: In SG, ALP activity was elevated 160% by 50 µm zinc, 140% by 7.5 µm curcumin, and 113% by 10 µm carsonic acid. SG had an induced expression of *ALP*, *RUNX2,* and *osteonectin* in nonosteogenic maintenance medium.
Chen et al., 2020 [76]	Murine primary OBs	SG1: 48 h RPM + R-irisin SG2: 48 h RPM CG: normal gravity	Lower doses of R-irisin promote both expression of *Collα1* and *ALP* (*p* < 0.05), activity of ALP, and calcium deposition in OBs. R-irisin also recovered the microgravity-induced reduction of *ALP* and *Collα1* (*p* < 0.001), ALP activity, and β-catenin expression.
Ethiraj et al., 2020 [77]	RAW264.7 pre-OCs	SG1: 24 h RCCS + MG-132 SG2: 24 h RCCS CG1: normal gravity CG2: normal gravity + MG-132	The proteasome inhibitor MG-132 in SG1 suppressed receptor activator of nuclear factor κβ receptor expression, compared to CG. MG-132 treatment in SG1 also showed a significant decrease in resorbed bone area compared to SG2.
Diao et al., 2018 [72]	OBs from newborn rat	SG1: 72 h RPM + polyphenols SG2: 72 h RPM CG: normal gravity	Polyphenols promoted ALP activity in SG1 compared to SG2 (*p* < 0.01). Polyphenols had a dose–effect response, but were still decreased compared to CG (*p* < 0.05).
He et al., 2020 [73]	Murine pre-OB cell line MC3T3-E1	SG1: 96 h RB SG2: 96 h RB + IL-6 mAb CG: normal gravity	IL-6 mAb increased ALP activity, osteoprotegerin level, and mRNA expression of *ALP*, *osteopontin* and *RUNX2*, while RANKL decreased in SG2 compared to SG1 (*p* < 0.05). All these factors were normalized compared to CG.
He et al., 2020 [73]	Macrophage cell line RAW264.7	SG1: 96 h RB SG2: 96 h RB + IL-6 mAb CG: normal gravity	IL-6 mAb decreased mRNA expression of cathepsin K and TRAP5 in SG2 compared to SG1 (*p* < 0.05). All these factors were normalized compared to CG.

Abbreviations: OC: osteoclast, OB: osteoblast, EC: endothelial cell, SG: study group, ISS: International Space Station, R-irisin: recombinant irisin, CG: control group, RUNX2: RUNX family transcription factor 2, Collα1: collagen type 1 α-1, bMSC: bone mesenchymal stem cell, SCHN: strontium-containing hydroxypatitie nanoparticles, ALP: alkaline phosphatase, *n*: number of individuals, HDBR: head-down tilt bed rest, LE: locomotor exercise, BMD; bone mineral density, BR: bed rest, RE: resistive exercise, PBD: pulse-based diet, CD: cognitive training, and diet protein and alkaline, HLU: hindlimb-unloading, PC-I: pan-caspase inhibitor, RANKL: receptor activator of nuclear factor κβ ligand, P1NP: N-terminal propeptide of type 1 procollagen, KOC: knockout casein kinase 2-interacting protein-1, CDL: constrained dynamic loading, BV/TV: bone volume fraction, TC: tibial compression, SMF: static magnetic field, GMF: geomagnetic field, TRAP5: tartrate-resistant acid phosphatase 5b, CEFFE: cell-free fat extract, PBS: phosphate-buffered saline, CMS: cyclic mechanical stretch treated bMSCs-derived exosomes, STE: static cultured bMSCs-derived exosomes, IL-6 mAb: interleukin-6 monoclonal antibody, ME: mechanical properties, RPM: random positioning machine, RCCS: rotary cell culture system, RB: rotary wall vessel bioreactor, RHLI: right hindlimb immobilization, ALF: alfacalcidol, ZOL: zoledronic acid.

**Table 3:** An overview of a selection of the latest clinical trials of µ*g*-induced BL and countermeasures.

Title and Identification Number	Subjects	Design	Outcome	Status/Conclusion
The Effects of Whole Body Unloading on Physiological Function (NCT03195348)	12	IV SG	Investigate the effects of hyper-buoyancy flotation in 7 days on skeletal muscles and bone mineral density.	Completed. No results posted yet.
A New Nutritional Countermeasure to Prevent the Deconditioning Induced by 60 Days of Antiorthostatic Bed Rest (NCT03594799)	20	IV RP Investigator masking	Investigate if XXS-2A-BR2 prevents or reduces the harmful effects caused by physical inactivity through 60 days of bed rest. Secondary outcome is the change in urinary C-telopeptide of type I collagen.	Completed. No effect on urinary C-telopeptide of type I collagen, serum β-C-telopeptide of type I collagen, NTX, alkaline phosphatase, P1NP, or osteocalcin.
Thigh Cuffs to Prevent the Deconditioning Induced by 5 Days of Dry Immersion (NCT03915457)	20	IV RP No masking	Investigate if thigh cuffs prevent or reduce the deconditioning caused by dry immersion. The outcome is the change in the balance of bone remodeling markers.	Completed. No results posted yet.
Planetary Habitat Simulation: Bone Metabolism Studies (NCT02637921)	14	IV RC Open-label masking	Investigate the effect of hypoxia and bed rest on bone metabolism. Outcomes include the change in markers of bone cell activity.	Completed. Serum calcium and NTX increased, while P1NP decreased. No difference between normoxia or hypoxia.
Understanding the Negative Effects of Bed Rest and Using Exercise as Countermeasure (NCT04964999)	24	IV RP Open-label masking	Investigate if exercise counteracts the negative effects caused by 2 week head-down tilt bed rest on bone markers among others.	Recruiting.
Integrative Study of Physiological Changes Induced by a 5-Day Dry Immersion on 20 Healthy Female Volunteers (NCT05043974)	20	IV SG Open-label masking	Investigate the changes caused by dry immersion for 5 days in female physiology. The outcome is the change in bone metabolism and bone mineral density.	Recruiting.

Abbreviations: NCT: national clinical trial, IV: interventional, SG: single-group assignment, RP: randomized parallel assignment, NTX: urinary N-telopeptide of type I collagen, P1NP: N-terminal propeptide of type 1 procollagen, RC: randomized crossover assignment.

## Data Availability

Not applicable.

## Abbreviations

AACE	American Association of Clinical Endocrinologists
ALF	Alfacalcidol
ALP	Alkaline phosphatase
AO	Antioxidants
ARED	Advanced resistive exercise device
BP	Bisphosphonate
BF	Bone formation
BL	Bone loss
BR/BRS	Bone resorption
BMD	Bone mineral density
BT	Bone turnover
bMSC	Bone mesenchymal stem cell
BV/TV	Bone volume fraction
CM	Countermeasure
CMS	Cyclic mechanical stretch treated bone mesenchymal stem cell-derived exosomes
DM	Differentiation marker
HLU	Hindlimb unloading
IL-6 mAb	Interleukin-6 monoclonal antibody
ISS	International Space Station
MP	Menopausal
OB	Osteoblast
OC	Osteoclast
OP	Osteoporosis
PC-I	Pan-caspase inhibitor
PI	Proteasome inhibitor
PMP	Postmenopausal
PTH	Parathyroid hormone
RANKL	Receptor activator of nuclear factor κβ ligand
RHLI	Right hindlimb immobilization
R-irisin	Recombinant irisin
SCHN	Strontium-containing hydroxyapatite nanoparticles
SERM	Selective estrogen receptor modulator
S-µ*g*	Simulated microgravity
uCaV	Urinary calcium excretion
uNTx	Urinary N-telopeptide
Wnt	Wingless-related integration site
ZOL	Zoledronic acid
µ*g*	Microgravity
